# Medicines Received

**Published:** 1868-11

**Authors:** 


Medicines Received.—We are indebted to Iridell & Co., of Philadelphia, for a box of Fluid Extracts, containing specimens of most of the important medicines in common use. They are prepared by an improved process, and represent their own weight of crude drug, of prime quality. So far as we are able to judge, they are of reliable purity and strength, and will be convenient for prescription, on account of their uniformity of composition. These medicines were received through O. H. P. Champlin & Co. who are agents for their sale in Buffalo.

